# All-cause and cause-specific mortality in psoriasis patients: a systematic review and meta-analysis

**DOI:** 10.3389/fimmu.2025.1610499

**Published:** 2025-07-24

**Authors:** Yi Yang, Qin Zhang, Anning Huang, Jinpeng Zhao, Jianren Yang, Lulu Wang, Guomei Xu

**Affiliations:** ^1^ Graduate School of Beijing University of Chinese Medicine, Beijing University of Chinese Medicine, Beijing, China; ^2^ Department of Dermatology, Beijing University of Chinese Medicine Third Affiliated Hospital, Beijing, China

**Keywords:** psoriasis, mortality, cardiovascular, infection, suicide, meta-analysis, hazard ratio

## Abstract

**Objective:**

The objective of this meta-analysis is to assess the all-cause and cause-specific mortality in patients with psoriasis.

**Method:**

In accordance with PRISMA guidelines, a systematic search of PubMed, EMBASE, and the Cochrane Library (from inception to March 2025) was conducted. Eligible studies comprised English-language cohort studies comparing mortality risk (HR/OR/RR) in adults with psoriasis versus healthy/non-psoriasis controls. Two reviewers independently screened studies, extracted data, and assessed study quality using the Newcastle-Ottawa Scale. Hazard ratios (HRs) were synthesized using random-effects models in Stata 14.0. Sensitivity analyses, subgroup analyses, and assessments of publication bias (via funnel plots and Egger’s test) were also performed.

**Result:**

A total of 20 studies involving 8825989 participants were included. Psoriasis patients demonstrated significantly increased risks of all-cause mortality [HR=1.19, 95% CI (1.11–1.28), P=0.000], cardiovascular mortality [HR = 1.32, 95% CI (1.11–1.58), P = 0.002], infection-related mortality [HR=1.24, 95% CI (1.13–1.36), P=0.000], and suicide mortality [HR=1.50, 95% CI (1.03–2.19), P=0.034]. The risk of mortality due to neoplasms was marginally elevated but not statistically significant [HR=1.05, 95% CI (0.98–1.12), P=0.151]. No significant associations were found for neurological disease mortality [HR=0.96, 95%CI (0.83–1.11), P=0.976] or accident-related mortality [HR=0.91, 95% CI (0.81–1.02), P=0.629]. Sensitivity analysis supports the findings. Subgroup analyses revealed higher all-cause mortality risks in Europe (HR=1.11) and Asia (HR=1.23), as well as an increased risk with greater disease severity (moderate-to-severe: HR=1.44; severe: HR=1.54). No publication bias was detected.

**Conclusion:**

Psoriasis is associated with an increased risk of all-cause, cardiovascular, infection-related, and suicide mortality, highlighting the need for enhanced monitoring and targeted interventions to prevent adverse outcomes particularly for individuals with severe psoriasis.

**Systematic review registration:**

https://www.crd.york.ac.uk/PROSPERO/view/CRD420251017192, identifier CRD420251017192.

## 1 Introduction

Psoriasis is a chronic, immune-mediated, relapsing inflammatory disorder characterized by erythematous plaques with silvery scales, it can present at any age, affecting approximately 125 million global population (1). Psoriasis is commonly associated with a range of systemic comorbidities, including cardiovascular diseases, malignancies, and infections, and imposes a substantial psychological burden due to its chronic and recurrent nature (2). Current therapeutic approaches include topical treatments, conventional systemic therapies, biologics targeting specific cytokines, and phototherapy (3). However, significant clinical gaps remain, as these therapies often exhibit high recurrence rates and suboptimal long-term efficacy (4).

Recent evidence underscores an elevated risk of all-cause and cause-specific mortality in psoriasis patients, largely driven by chronic inflammation and immune dysregulation (5, 6). Proinflammatory cytokines, such as TNF-α and IL-17, contribute to the acceleration of atherosclerosis, thereby increasing cardiovascular risk, while systemic immunosuppression enhances susceptibility to severe infections (7). Additionally, psychosocial stressors associated with visible skin lesions substantially elevate the incidence of anxiety, depression and suicides (8). These findings highlight the urgent need for integrated management strategies that address both the cutaneous manifestations and systemic complications of psoriasis.

To more accurately assess the all-cause and cause-specific mortality risks associated with psoriasis, we conducted a systematic review and meta-analysis integrating cohort studies that evaluated the impact of psoriasis on mortality outcomes, with the aim of informing targeted interventions and improving clinical monitoring of high-risk subgroups.

## 2 Method

This study adhered to the Meta-analysis of Preferred Reporting Items for Systematic Reviews and Meta-Analyses(PRISMA) (9) guidelines and followed a pre-registered protocol on the International Prospective Register of Systematic Reviews (PROSPERO) platform, under approval number CRD420251017192.

### 2.1 Data sources

A comprehensive systematic search was conducted across the PubMed, EMBASE, and Cochrane Library databases using relevant Medical Subject Heading (MeSH) terms for PubMed, along with appropriate keywords. The search covered articles published from the inception of each database up to March 2025. Key search terms included “Psoriasis,” “Mortality,” “Risk,” and their relevant synonyms. The complete search strategy is outlined in **Supplementary Material Table 1**.

### 2.2 Inclusion criteria

Studies were included based on the following criteria: (1) prospective or retrospective cohort study design; (2) patients diagnosed with psoriasis, aged 18 years or older, regardless of disease duration or nationality; (3) a control group consisting of healthy individuals or non-psoriasis patients; (4) the primary outcome of mortality risk, reported as hazard ratio (HR), or odds ratio (OR), relative risk (RR) during the follow-up period; (5) studies published in English.

### 2.3 Exclusion criteria

The following studies were excluded: (1) duplicate publications; (2) reviews, clinical case reports, meeting abstracts, letters, or comments; (3) incomplete data or studies lacking outcomes of interest; (4) studies focused on hospitalized patients; (5) studies investigating alcohol-related mortality.

### 2.4 Study selection

Two authors (Yi Yang and Qin Zhang) independently screened all identified studies, and the results were compared. If the results were consistent, the final analysis was conducted. In cases of disagreement, the full-text articles were reviewed to ensure the studies met the eligibility criteria. Any discrepancies were resolved through group discussions.

### 2.5 Data extraction

A data extraction table was created using Microsoft Excel. Both authors (Yi Yang and Qin Zhang) independently extracted relevant data from the eligible studies, including the first author, publication year, country, study type, number of events, and confounding factors. The extracted data were cross-checked for accuracy, and discrepancies were resolved through group discussions to ensure consistency.

### 2.6 Quality assessment

The quality of the included studies was assessed using the Newcastle-Ottawa Scale (NOS) (10), which evaluates studies based on three domains: selection, comparability, and outcome. The NOS score ranges from 0 to 9, with higher scores indicating better study quality. The criteria include participant selection (4 points), comparability between groups (2 points), and assessment of exposure factors (3 points). Studies were classified as high quality (NOS ≥ 7), medium quality (NOS 4–6), or low quality (NOS 0–3).

### 2.7 Data analysis

Data analysis was performed using Stata software (version 14). Mortality risk was reported as HR with corresponding 95% confidence intervals (CIs). Depending on the results of the heterogeneity test, either a random-effects or fixed-effects model was used. A P-value < 0.1 or I² > 50% indicated high heterogeneity. Given the potential for clinical, methodological, and statistical heterogeneity, a random-effects model was typically applied for the meta-analysis (11, 12). Sensitivity analysis was conducted to assess the robustness of the results, with a one-by-one elimination method used to explore sources of heterogeneity. Subgroup analyses were performed based on cohort study type, study region, and the severity of psoriasis. Publication bias was assessed using funnel plots and Egger’s regression tests.

## 3 Results

### 3.1 Literature search

**Figure 1:**
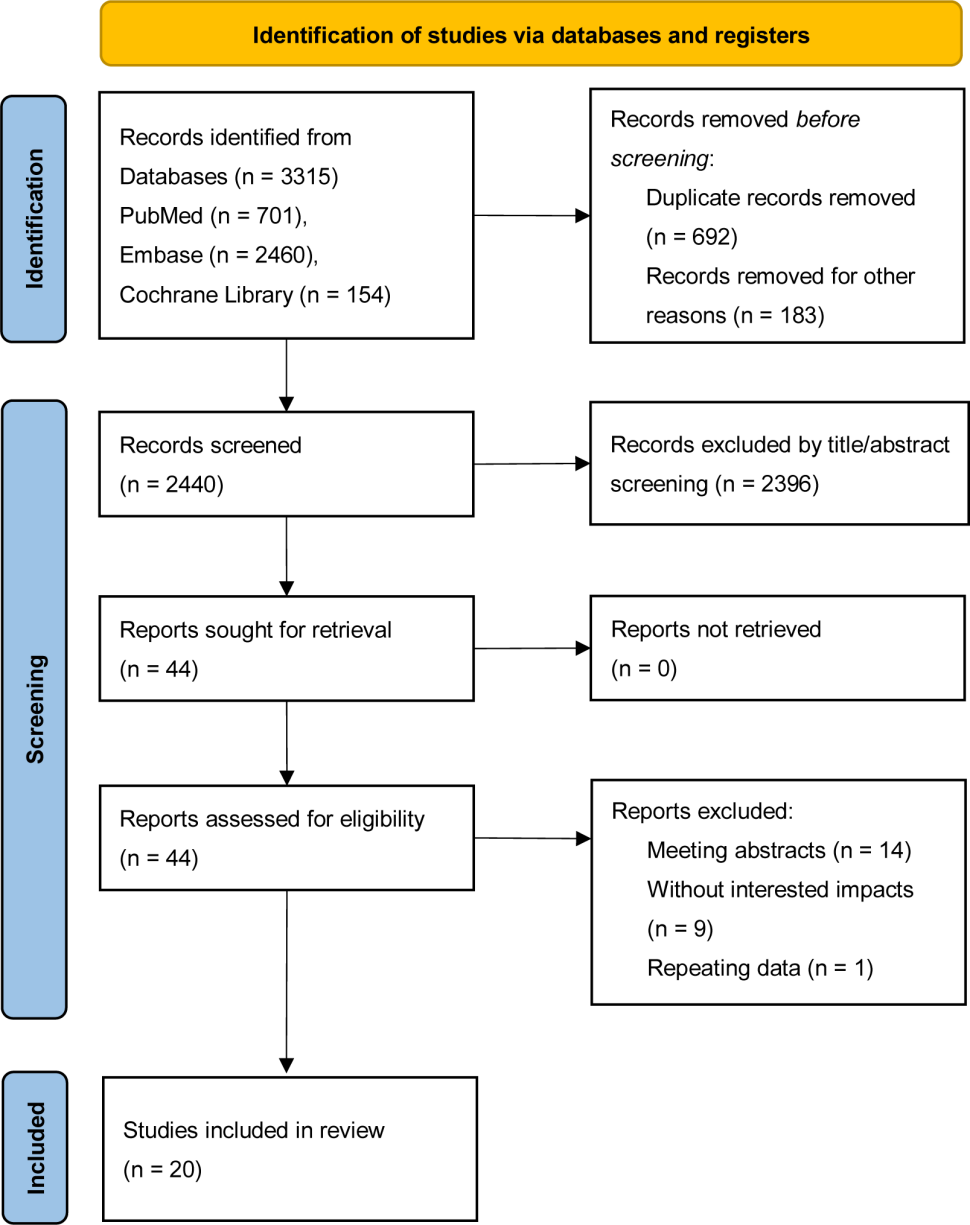
Literature screening.

A preliminary literature search identified a total of 3,315 relevant records, including 701 articles from PubMed, 2,460 from EMBASE, and 154 from the Cochrane Library. These records were imported into EndNote reference management software. After removing duplicates, 2,623 records remained. Following a review of the titles and abstracts, irrelevant records were excluded, leaving 44 articles for full-text review. Any uncertain records were assessed by reading the full text to verify their eligibility for inclusion in the study. A total of 20 cohort studies (5, 6, 13–30) were deemed eligible after this screening process, and the flow of literature screening is illustrated in **Figure 1**.

### 3.2 Basic characteristics

The 20 cohort studies included a total sample size of 8825989 participants, comprising 851942 psoriasis patients and 7974047 controls. The baseline characteristics of the included participants are summarized in **Table 1**. Among the studies, 15 (5, 6, 13–15, 17–19, 21–25, 29, 30) focused on the risk of all-cause mortality in psoriasis patients, and 7 (5, 14–16, 20, 22, 28) studies evaluated the risk of cardiovascular mortality. Mortality related to infection, neoplasms, and suicide was assessed in 4 studies (14–16, 21, 26, 28) each. Additionally, 3 studies (14, 16, 21) examined mortality due to neurological diseases, and 3 (14, 21, 28) others explored accident-related mortality. The studies included were published between 2007 and 2024. The adjusted confounding factors varied across studies, with age, gender, and hypertension being the most commonly controlled variables. Of the 20 studies, 10 were conducted in Europe, 7 in America, and 4 in Asia. **Table 1** presents the detailed characteristics of the cohort studies.

**Table 1:** Characteristics of studies included in the meta-analysis.

Author	Year	Region	Study type	Sample size	Cause of mortality	Confounders adjusted
Si, Z (6).	2024	America	Retrospective cohort study	Total:14021, psoriasis:13664, no psoriasis:357	All-cause mortality	Sex, age group, race, education, marital status, BMI category, smoking status, drinking status, hypertension and diabetes
Abuabara, K (28).	2010	Europe	Retrospective cohort study	Total:17933, psoriasis:14330, no psoriasis:3603	Accidents, cardiovascular disease, chronic lower respiratory disease, dementia, diabetes, infection, kidney disease, liver disease, malignant neoplasms, other, suicide, unknown/missing	Age, sex
Ahlehoff, O (27).	2011	Europe	Retrospective cohort study	Total:4040257, psoriasis:4003265, no psoriasis:36992	All-cause mortality, cardiovascular disease, composite endpoint: stroke, myocardial infarction and cardiovascular death	/
Chen, T. C (26).	2024	Asia	Retrospective cohort study	Total:1298015, psoriasis:1112581, no psoriasis:185434	Any infection, respiratory infections, sepsis, skin/soft-tissue infections, urinary tract infections, infectious arthropathies, endocarditis, tuberculosis, hepatitis B, hepatitis C	Age, sex and comorbidities which are related to lifestyle factors, including chronic obstructive pulmonary disease, hyperlipidemia, hypertension, alcohol-related conditions, ischemic heart disease, hospital-diagnosed obesity, and type 2 diabetes and other covariates, including cancer, rheumatoid arthritis, psoriatic arthritis, dermatological immune-mediated inflammatory diseases, inflammatory bowel disease, multiple sclerosis, spondylopathies, systemic connective tissue disorders and inflammatory polyarthritis
Dai, Y. X (25).	2018	Asia	Retrospective cohort study	Total:213402, psoriasis:106701, no psoriasis:106701	All-cause mortality	Age, sex, socioeconomic status, residence, hypertension, diabetes mellitus, dyslipidemia, coronary artery disease, stroke, connective tissue disease, renal diseases, chronic liver diseases and cirrhosis and hepatitis, chronic obstructive pulmonary disease, and cancer
Gelfand, J. M (24).	2007	Europe	Retrospective cohort study	Total:712952, psoriasis:575433, no psoriasis:137519	All-cause mortality	Age, sex
Iskandar, I. Y. K (23).	2022	Asia	Retrospective cohort study	Total:1356020, psoriasis:1232717, no psoriasis:123303	All-cause mortality	Index year, age and sex
Kan, J (5).	2024	America	Prospective cohort study	Total:19741, psoriasis:19199, no psoriasis:542	All-cause mortality, CVD	Sex, age, race, education, marital status, family PIR, smoking and drinking
Kong, X. and W. Wang (22)	2024	America	Prospective cohort study	Total:11155, psoriasis:10865, no psoriasis:290	All-cause mortality, CVD	Age, sex, race/ethnicity, marital status, educational level, poverty-income ratio, smoking, drinking, physical activity, estimated glomerular filtration rate and urinary albumin/creatinine ratio
Lee, M. S (21).	2017	Asia	Prospective cohort study	Total:160414, psoriasis:80207, no psoriasis:80207	All-cause mortality, circulatory system diseases, malignancies, respiratory system diseases, infectious diseases, digestive system diseases, urogenital diseases, endocrine, nutritional and metabolic diseases, mental and behavioral disorders, diseases of the nervous system and sense organs, intentional self-harm or suicide, accidents and unintentional injuries, others	/
Mehta, N. N (20).	2010	Europe	Retrospective cohort study	Total:17933, psoriasis:14330, no psoriasis:3603	Cardiovascular disease	Age, sex, hyperlipidemia, hypertension, smoking, diabetes
Pezzolo, E (19).	2021	Europe	Retrospective cohort study	Total:61758, psoriasis:49065, no psoriasis:12693	All-cause mortality	/
Prodanovich, S (18).	2009	America	Retrospective cohort study	Total:5736, psoriasis:3236, no psoriasis:2500	All-cause mortality	Age, sex, hypertension, diabetes mellitus, dyslipidemia, tobacco, any vascular disease.
Semenov, Y. R (17).	2021	America	Retrospective cohort study	Total:13031, psoriasis:12684, no psoriasis:347	All-cause mortality	Age
Skov, L (16).	2019	Europe	Retrospective cohort study	Total:42096, psoriasis:29936, no psoriasis:12160	Immune mechanism, endocrine, nutritional and metabolic diseases, mental and behavioral disorders, diseases of the nervous system, diseases of the circulatory system (heart disease), other diseases of the circulatory system, diseases of the respiratory system, diseases of the digestive system, diseases of the musculoskeletal system and connective tissue, diseases of the genitourinary system, symptoms, signs and abnormal clinical and laboratory findings, not elsewhere classified, Injury, poisoning and certain other consequences of external causes, external causes of morbidity and mortality: intentional self-harm, unknown causes	Age, sex
Springate, D. A (30).	2017	Europe	Retrospective cohort study	Total:612898, psoriasis:508457, no psoriasis:104441	All-cause mortality	/
Stern, R. S. and A. Huibregtse (15).	2011	Europe	prospective cohort study	Total:2752, psoriasis:1376, no psoriasis:1376	All-cause mortality, major cardiovascular, diseases, neoplasms, other causes	Age, sex
Svedbom, A (14).	2015	Europe	Retrospective cohort study	Total:193849, psoriasis:154775, no psoriasis:39074	All-cause mortality, liver disease, missing, other causes, diabetes mellitus, kidney disease, cardiovascular disease, neoplasm, accidents, severe infection, suicide, CLRD, neurological disease	/
Zhao, H (13).	2024	America	prospective cohort study	Total:12107, psoriasis:11829, no psoriasis:278	All-cause mortality	Sex, age group, race, education, marital status, BMI category, smoking status, drinking status and diabetes
Zhou, T (29).	2025	America	prospective cohort study	Total:19919, psoriasis:19397, no psoriasis:522	All-cause mortality	Age, gender, race, education level, marital status, PIR, drinking, smoking, moderate activity, diabetes, hypertension, cardiovascular disease, LDL-C and total cholesterol

### 3.3 Quality assessment

The quality of all included studies was assessed using the Newcastle-Ottawa Scale (NOS), which evaluates sample selection, comparability, and exposure factors, as shown in **Table 2**. The average NOS score for the studies was 7.6, with scores ranging from 6 to 9. Specifically, 3 studies scored 6 points, 6 studies scored 7 points, 8 studies scored 8 points, and 3 studies scored 9 points. This indicates that the overall quality of the studies included in this meta-analysis is high.

**Table 2:** Newcastle-Ottawa quality of cohort studies.

Study	Year	Selection	Comparability	Outcome	Total
Cohort studies (n=20)					
Si, Z (6).	2024	****	**	***	9
Abuabara, K (28).	2010	***	**	**	7
Ahlehoff, O (27).	2011	****	*	**	7
Chen, T. C (26).	2024	****	**	**	8
Dai, Y. X (25).	2018	***	**	**	7
Gelfand, J. M (24).	2007	****	**	***	9
Iskandar, I. Y. K (23).	2022	****	**	**	8
Kan, J (5).	2024	***	**	*	6
Kong, X. and W. Wang (22)	2024	****	**	**	8
Lee, M. S (21).	2017	***	*	**	6
Mehta, N. N (20).	2010	****	**	**	8
Pezzolo, E (19).	2021	****	*	*	6
Prodanovich, S (18).	2009	****	**	**	8
Semenov, Y. R (17).	2021	****	**	***	9
Skov, L (16).	2019	****	**	*	7
Springate, D. A (30).	2017	****	*	***	8
Stern, R. S. and A. Huibregtse (15).	2011	****	**	*	7
Svedbom, A (14).	2015	****	*	**	7
Zhao, H (13).	2024	****	**	**	8
Zhou, T (29).	2025	****	**	**	8

### 3.4 Risk of all-cause mortality

**Figure 2:**
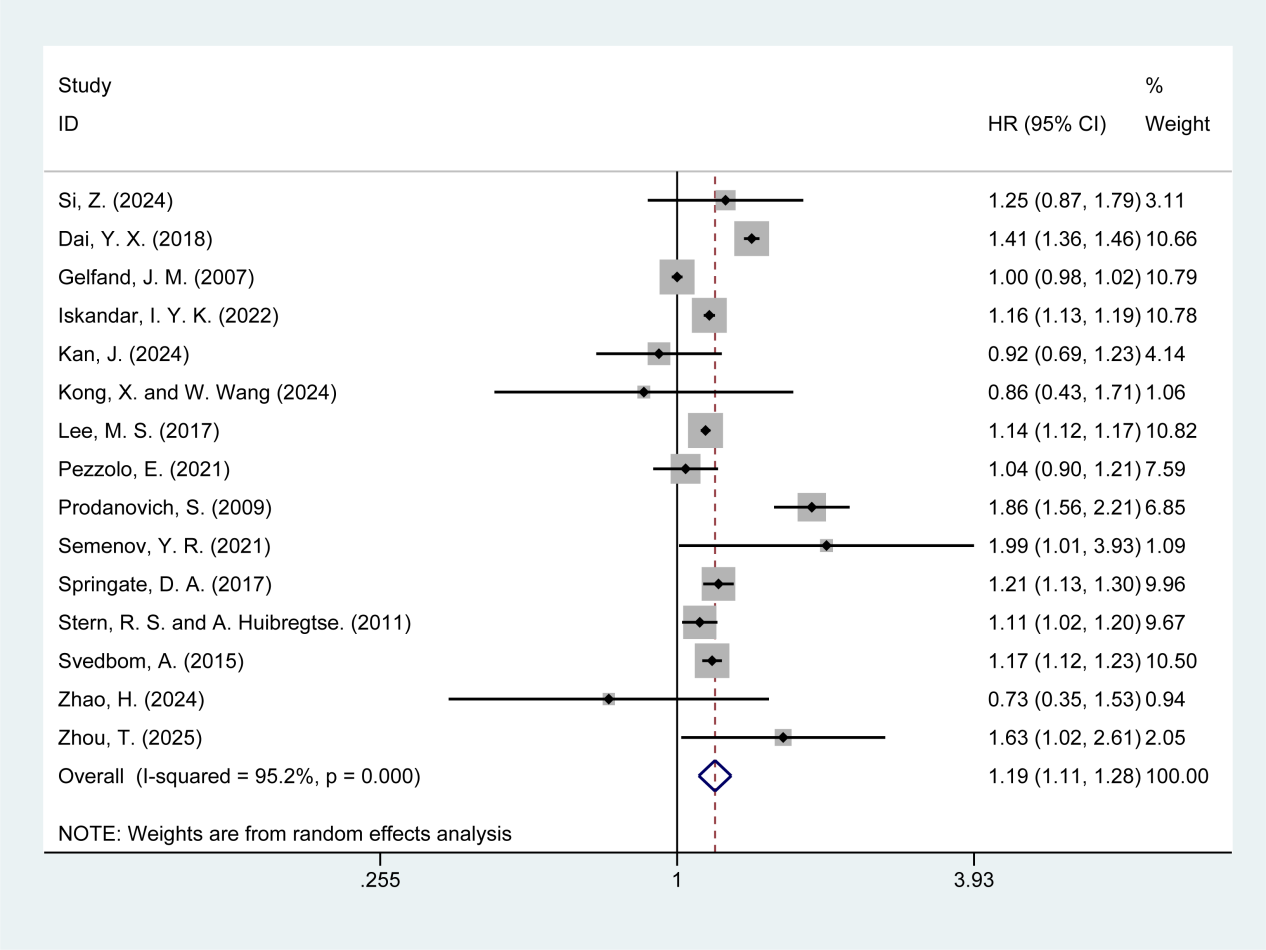
Forest plot for the risk of all-cause mortality in psoriasis.

Of the 20 studies included, 15 (5, 6, 13–15, 17–19, 21–25, 29, 30) reported the risk of all-cause mortality in psoriasis patients. The meta-analysis revealed that psoriasis patients had a significantly increased risk of all-cause mortality [HR = 1.19, 95% CI (1.11–1.28), I² = 95.2%, P = 0.000], with substantial heterogeneity (**Figure 2**). Further investigation into the sources of heterogeneity is warranted. Sensitivity analysis demonstrated the robustness of the results, as the exclusion of any individual study did not alter the overall findings (**Supplementary Material Figure 1**).

### 3.5 Risk of cardiovascular mortality

**Figure 3:**
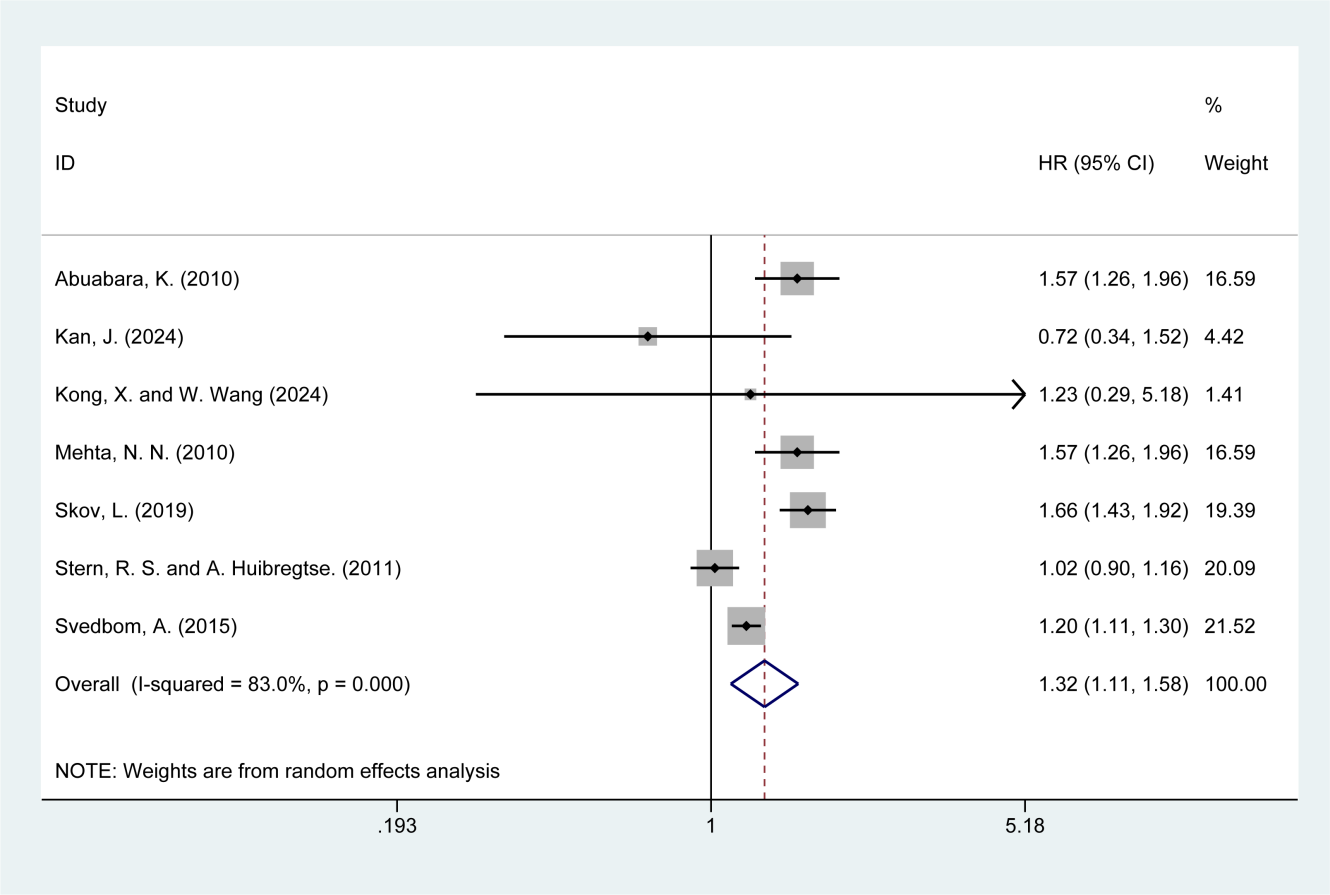
Forest plot for the risk of cardiovascular mortality in psoriasis.

Seven studies (5, 14–16, 20, 22, 28) reported the risk of cardiovascular mortality in psoriasis patients. The meta-analysis showed that psoriasis was associated with a higher risk of cardiovascular mortality [HR = 1.32, 95% CI (1.11–1.58), I² = 83%, P = 0.002, **Figure 3**]. Although sensitivity analysis confirmed the stability of the results, the high I² value (83%) and significant P-value (P = 0.000) from the Q-test indicate considerable heterogeneity. Both clinical and methodological heterogeneity were considered as potential sources, while statistical heterogeneity was ruled out. The results of the sensitivity analysis are presented in the **Supplementary Material Figure 2**.

### 3.6 Risk of infection mortality

**Figure 4:**
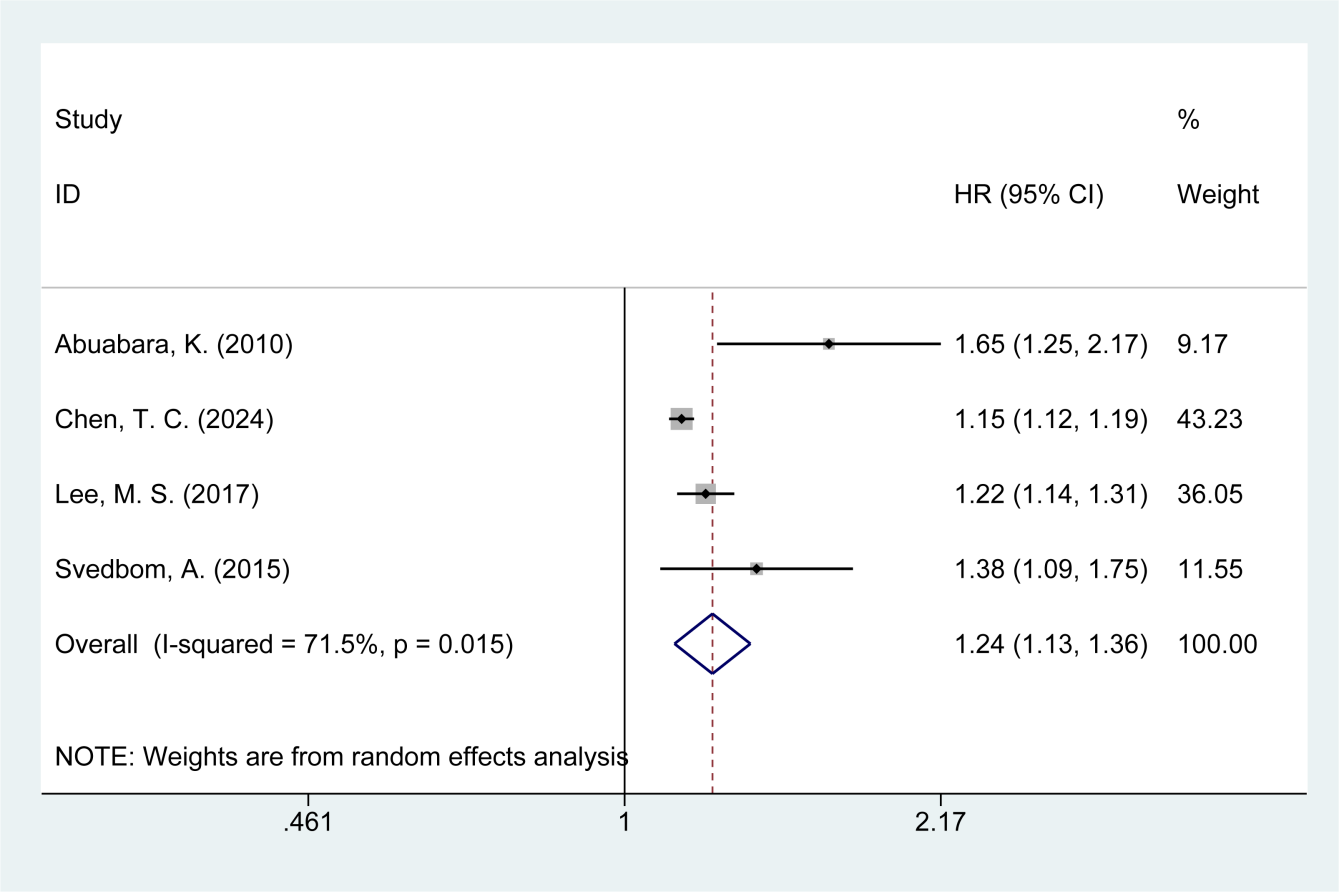
Forest plot for the risk of infection mortality in psoriasis.

Four studies (14, 21, 26, 28) assessed the risk of infection-related mortality in psoriasis patients. The meta-analysis revealed an increased risk of infection mortality among psoriasis patients [HR = 1.24, 95% CI (1.13–1.36), I² = 71.5%, P = 0.000, **Figure 4**]. Sensitivity analysis indicated that none of the studies reversed the pooled effect, supporting the reliability of the findings regarding infection mortality in psoriasis patients (**Supplementary Material Figure 3**).

### 3.7 Risk of neoplasm mortality

**Figure 5:**
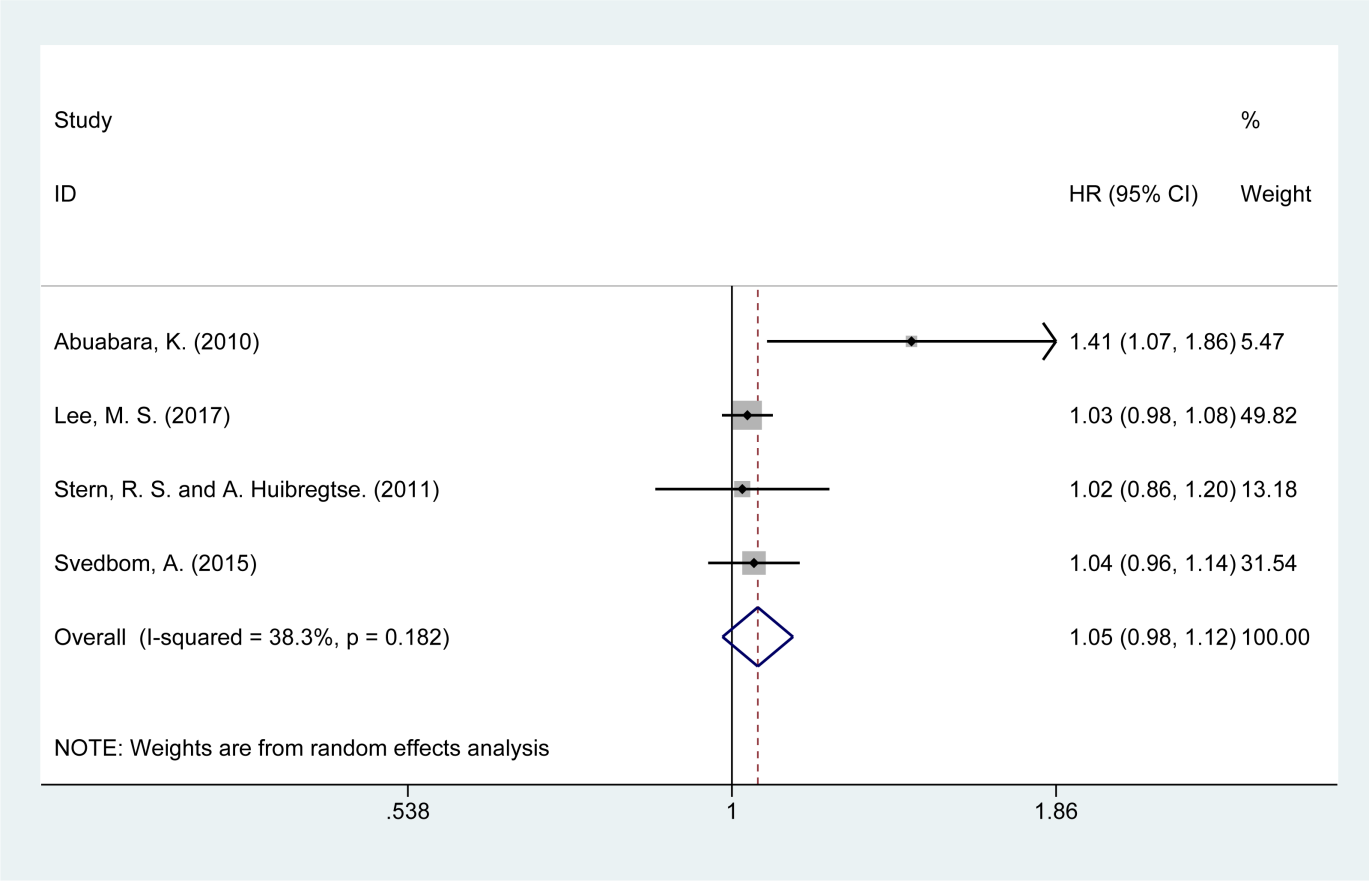
Forest plot for the risk of neoplasm mortality in psoriasis.

The risk of neoplasm-related mortality was analyzed in four studies (14, 15, 21, 28). The summary analysis showed a slight increase in the risk of mortality due to neoplasms [HR = 1.05, 95% CI (0.98–1.12), I² = 38.3%, P = 0.151, **Figure 5**]. Sensitivity analysis confirmed the stability of these results (**Supplementary Material Figure 4**).

### 3.8 Risk of suicide mortality

**Figure 6:**
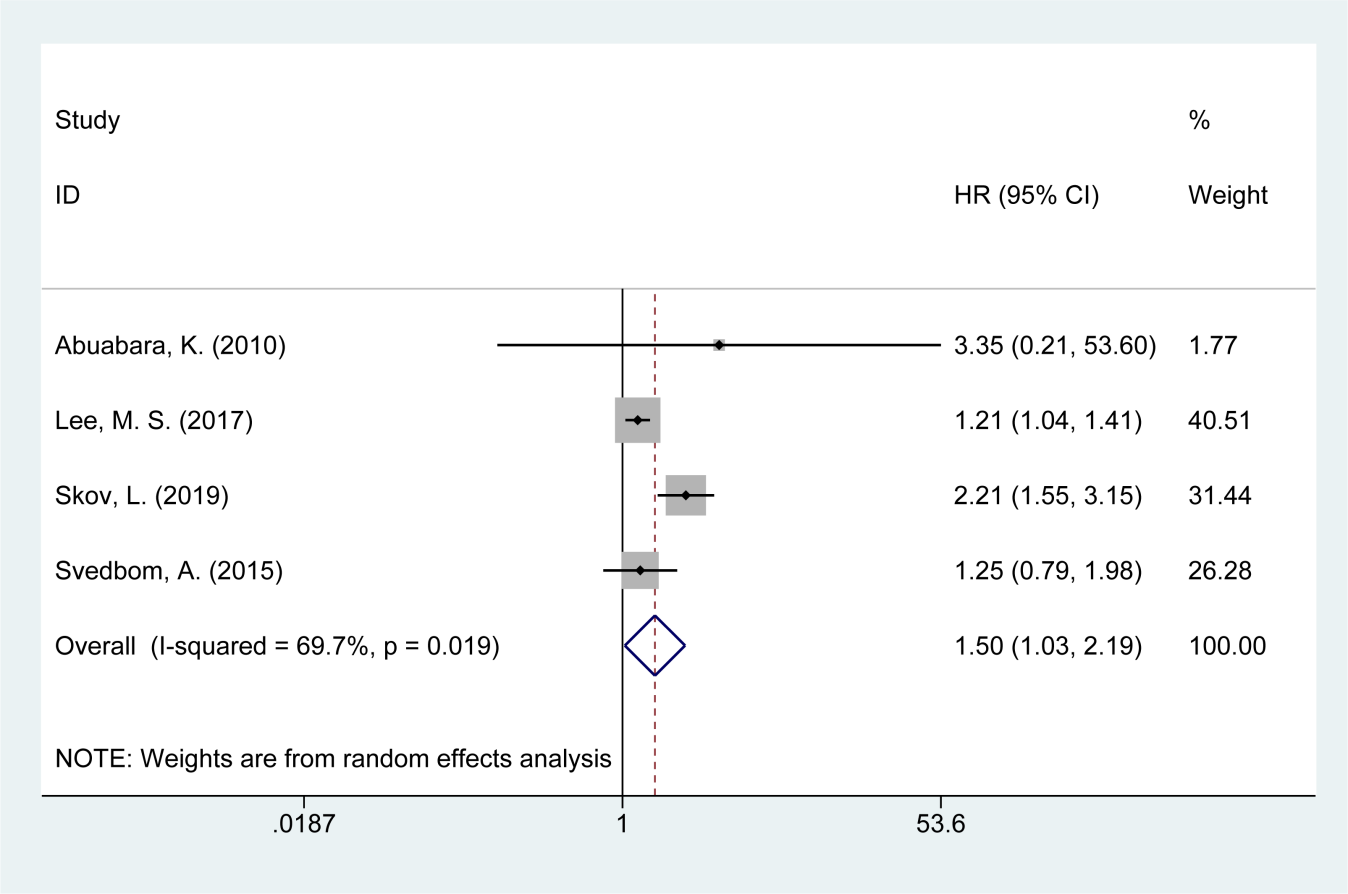
Forest plot for the risk of suicide mortality in psoriasis.

Four studies (14, 16, 21, 28) reported on the risk of suicide-related mortality in psoriasis patients. The meta-analysis found an increased risk of suicide mortality [HR = 1.50, 95% CI (1.03–2.19), I² = 69.7%, P = 0.034, **Figure 6**]. Sensitivity analysis confirmed that the results remained consistent and reliable (**Supplementary Material Figure 5**).

### 3.9 Risk of neurological disease mortality

**Figure 7:**
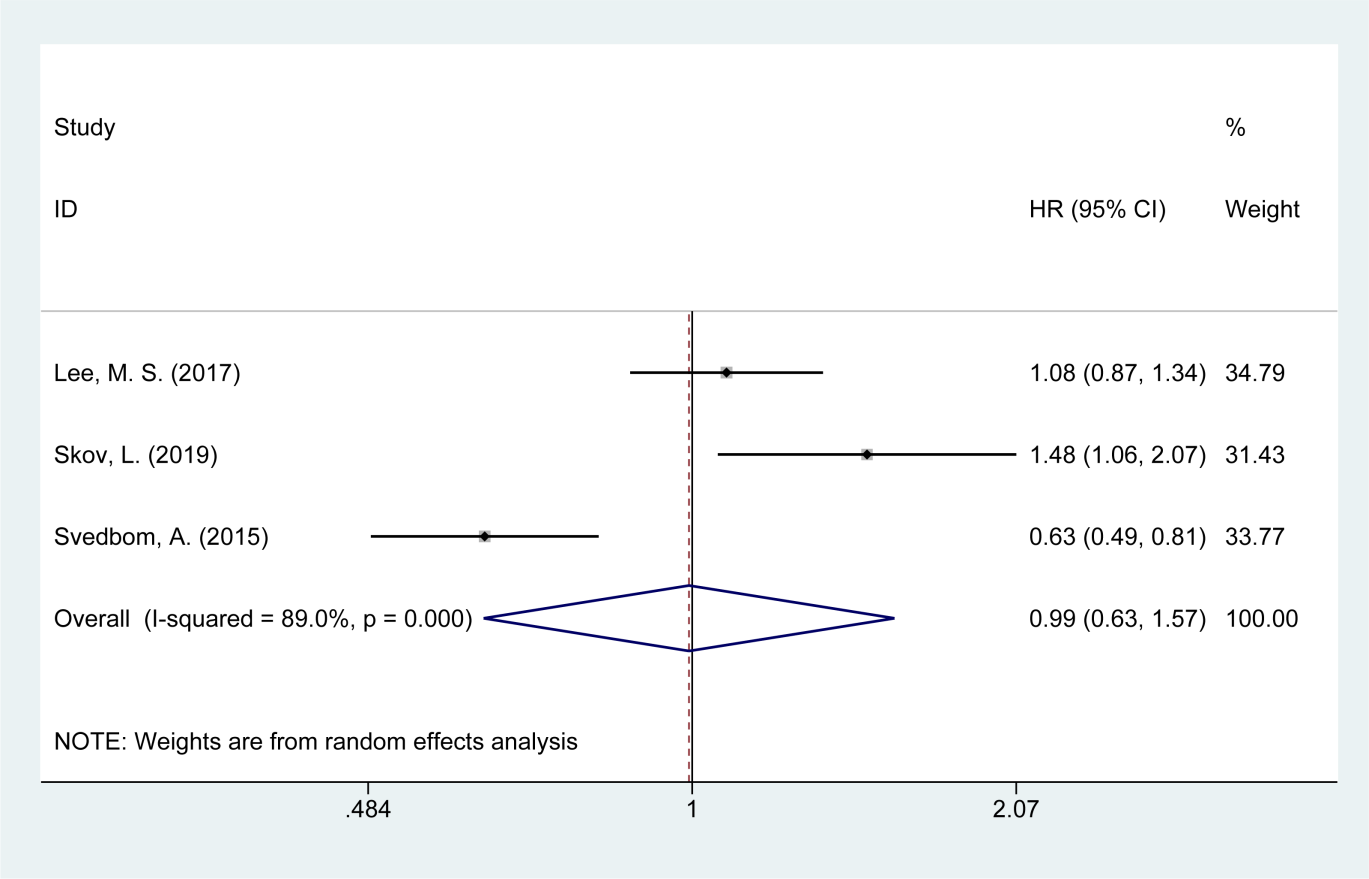
Forest plot for the risk of neurological disease mortality in psoriasis.

Three studies (14, 16, 21) evaluated the mortality risk from neurological diseases in psoriasis patients. The meta-analysis showed no significant increase in the risk of neurological disease-related mortality [HR = 0.96, 95% CI (0.83–1.11), I² = 89.0%, P = 0.976, **Figure 7**].

### 3.10 Risk of accident mortality

**Figure 8:**
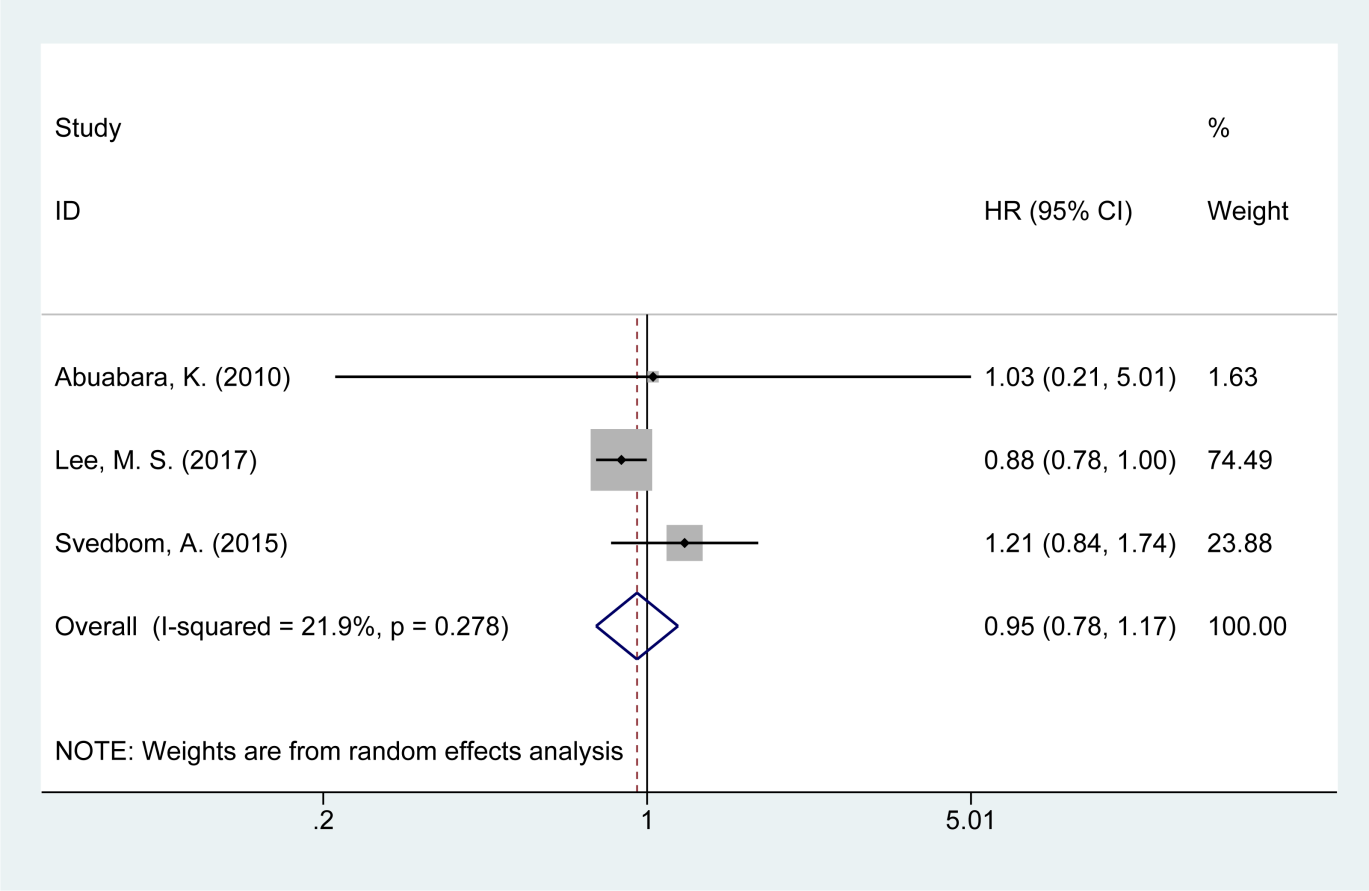
Forest plot for the risk of accident mortality in psoriasis.

Three studies (14, 21, 28) examined the risk of accident-related mortality in psoriasis patients. The meta-analysis found a slight increase in accident-related mortality risk [HR = 0.91, 95% CI (0.81–1.02), I² = 21.9%, P = 0.629, **Figure 8**].

### 3.11 Subgroup analysis

Subgroup analyses were conducted to investigate the risk of all-cause mortality based on study type, region, and psoriasis severity. The results indicated that the risk of all-cause mortality was significantly higher in studies conducted in Europe [HR = 1.107, 95% CI (1.006–1.217), I² = 92.9%, P = 0.036] and Asia [HR = 1.230, 95% CI (1.097–1.379), I² = 98.1%, P = 0.000], but not in studies from America. Additionally, psoriasis severity was found to be positively associated with an increased risk of all-cause mortality. Specifically, moderate-to-severe psoriasis [HR = 1.435, 95% CI (1.174–1.755), I² = 0.0%, P = 0.000] and severe psoriasis [HR = 1.543, 95% CI (1.441–1.652), I² = 67.6%, P = 0.000] were associated with higher mortality risk, whereas mild psoriasis did not show a significant association. The detailed findings are shown in **Table 3**.

**Table 3:** Results of subgroup analysis.

Subgroup	All-Cause Mortality	
	Pooled hazard ratio (95% CI)	*P*
Study type		
Retrospective	1.240 (1.114-1.381)	0.000
Prospective	1.121 (1.055-1.192)	0.000
*Region*		
America	1.276 (0.938-1.736)	0.120
Europe	1.107 (1.006-1.217)	0.036
Asia	1.230 (1.097-1.379)	0.000
Disease severity		
Mild	1.086 (0.957-1.233)	0.200
Moderate to severe	1.435 (1.174-1.755)	0.000
Severe	1.543 (1.441-1.652)	0.000

### 3.12 Publication bias

**Figure 9:**
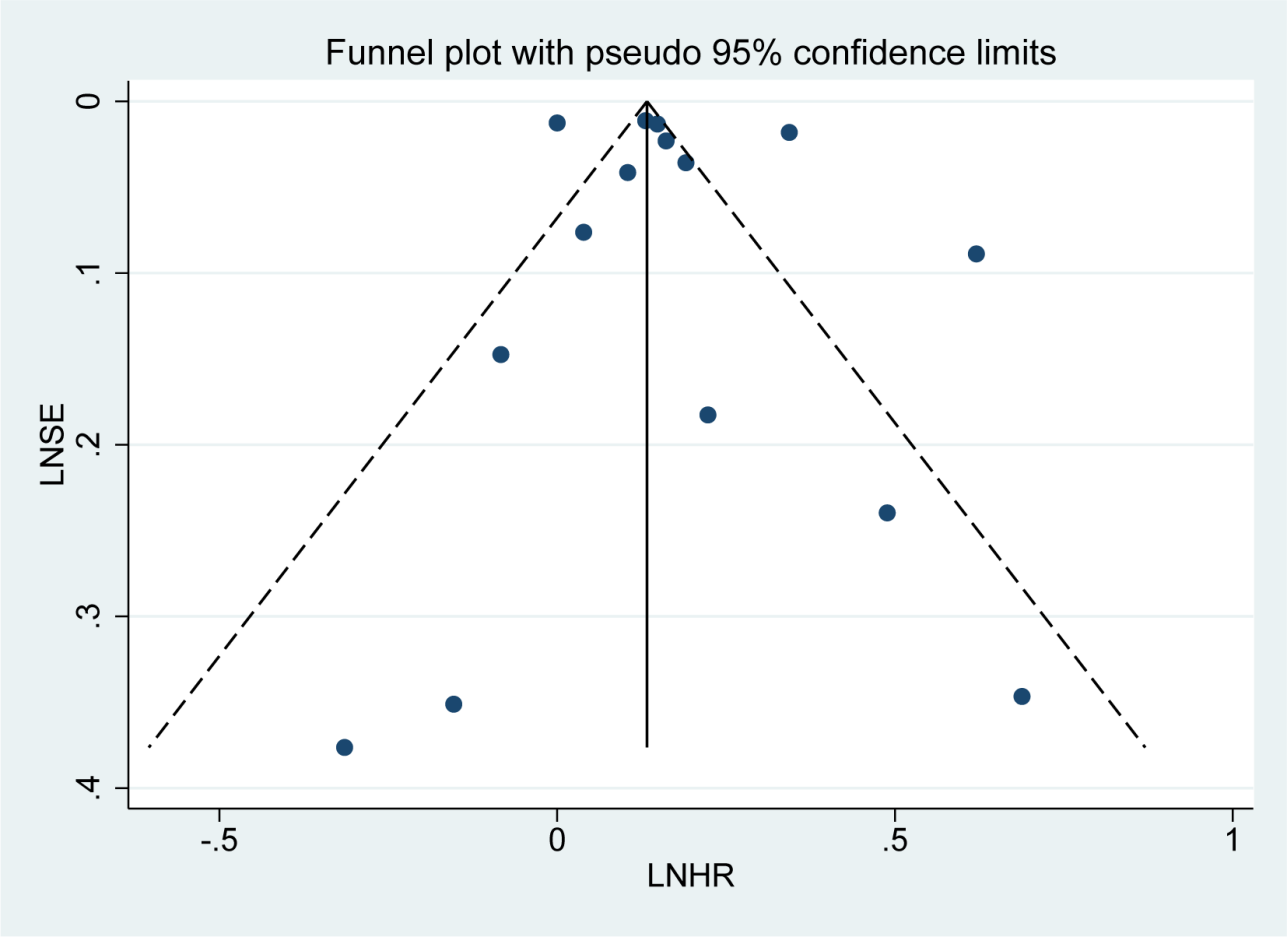
Funnel plot for all-cause mortality in psoriasis patients.

Publication bias was assessed for the 15 studies on overall mortality risk using a funnel plot. The subsequent bias test revealed no significant publication bias (P = 0.518 > 0.1, **Figure 9**), and the funnel plot exhibited a roughly symmetrical distribution, suggesting that the results are less likely to be influenced by publication bias.

## 4 Discussion

### 4.1 Main findings

This meta-analysis, which includes 20 cohort studies with a total of 8,825,989 participants, reveals a significant association between psoriasis and an increased risk of all-cause mortality (HR=1.19, 95% CI: 1.11–1.28) as well as cause-specific mortality. The latter includes heightened risks for cardiovascular (HR=1.32), infection-related (HR=1.24), and suicide-related mortality (HR=1.50). However, no significant associations were found for mortality related to neurological diseases, or accidents. Notably, the risk of all-cause mortality escalates with disease severity, with moderate-to-severe psoriasis showing an HR of 1.44 and severe psoriasis an HR of 1.54, indicating a potential dose-response relationship. Subgroup analysis revealed significant regional disparities in all-cause mortality risk, with HR of 1.107 (Europe) and 1.230 (Asia). These findings support the notion that the systemic inflammatory burden of psoriasis may exacerbate comorbid conditions and contribute to elevated mortality risks.

### 4.2 Interpretation of findings

Previous studies have demonstrated that psoriasis increases the risk of all-cause mortality, with subgroup analyses revealing a particularly elevated risk in patients with severe psoriasis (31). However, the association between psoriasis and all-cause mortality has not been systematically examined. In contrast, our analysis incorporates recent cohort studies and conducts subgroup analyses stratified by study design (retrospective/prospective) and geographic region (America/Europe/Asia), significantly enhancing the reliability of findings regarding psoriasis-associated all-cause mortality. Earlier systematic reviews identified elevated risks of mortality from cardiovascular, hepatic, renal, infectious, neoplastic, and lower respiratory diseases in psoriasis patients but failed to detect an increased risk of suicide-related mortality, likely due to limited statistical power from small sample sizes with only 12 relevant studies included (31). Notably, the present study analyzed more recent and larger-scale cohorts and revealed a significantly elevated suicide mortality risk, underscoring the critical role of psychosocial burden in psoriasis-related mortality.

### 4.3 Underlying mechanisms

The elevated mortality risks observed in psoriasis patients are driven by multifaceted pathophysiological mechanisms. Chronic inflammation mediated by the Th17/IL-23/IL-17 axis promotes endothelial dysfunction and accelerates atherosclerosis through elevated levels of TNF-α, IL-17, and IL-6, which enhance vascular endothelial activation and plaque instability (32). Concurrently, for patients with psoriasis, systemic immunosuppression from biologics and inherent immune dysregulation heightening susceptibility to severe infections such as sepsis and respiratory infections (33, 34). Psychosocial burdens arise from visible skin lesions, which induce social stigmatization and chronic stress. Proinflammatory cytokines cross the blood-brain barrier, disrupting serotonin and dopamine pathways via microglial activation and synaptic dysfunction, thereby amplifying suicide risk (35). The regional disparities in all-cause mortality risk may reflect interactions between environmental factors and genetic susceptibility (36).

### 4.4 Clinical and preventive implications

This meta-analysis revealed that patients with psoriasis face an increased risk of all-cause mortality and elevated cause-specific mortality risks, including cardiovascular diseases, infections, and suicide. These findings underscore the necessity of prioritizing daily management and prevention of comorbidities in psoriasis patients to mitigate mortality risks. For preventive strategies, regular screening for hypertension, dyslipidemia, atherosclerosis, and infection susceptibility is recommended to address cardiovascular and infectious risks (7). Additionally, routine psychological assessments and targeted mental health interventions are critical for patients exhibiting suicidal ideation, particularly given the psychosocial burden associated with visible skin lesions (37). Subgroup analysis demonstrated that patients with severe psoriasis faced significantly higher mortality risks. This highlights the imperative for stratified management based on disease severity, including tailored interventions such as intensified cardiovascular monitoring, optimized biologic dosing, and multidisciplinary mental health support for high-risk subgroups (38).

### 4.5 Implications and limitations

This study possesses several strengths, including a large sample size, rigorous adherence to PRISMA guidelines, evaluation of bias risk, sensitivity analyses, and subgroup analyses stratified by study type, region, and disease severity. However, some limitations must be acknowledged. First, only cohort studies were included, which controlled for confounding factors to some extent; however, certain subgroups had a limited number of studies, which reduced statistical power. Future research could broaden the inclusion criteria to incorporate additional study designs. Second, significant heterogeneity was observed across studies, including variations in the definition of severe psoriasis, classification of specific causes of death, follow-up duration, and adjustment for confounding factors. To account for potential heterogeneity, a random-effects model was applied. These limitations should be considered when interpreting the findings of this study.

## 5 Conclusions

This meta-analysis revealed that psoriasis is associated with an elevated risk of all-cause mortality, as well as increased mortality attributable to cardiovascular diseases, infections, suicide, and other factors. Our findings underscore the necessity of multidisciplinary interventions, particularly in severe cases. Future studies should further elucidate the underlying pathophysiological mechanisms to facilitate the development of more effective preventive strategies and therapeutic approaches for patients with psoriasis.

## Data Availability

The original contributions presented in the study are included in the article/**Supplementary Material**. Further inquiries can be directed to the corresponding author.

## References

[1] Armstrong AW Read C . Pathophysiology, clinical presentation, and treatment of psoriasis: A review. Jama. (2020) 323:1945–60. doi: 10.1001/jama.2020.4006, PMID: , PMID: 32427307

[2] Bu J Ding R Zhou L Chen X Shen E . Epidemiology of psoriasis and comorbid diseases: A narrative review. Front Immunol. (2022) 13:880201. doi: 10.3389/fimmu.2022.880201, PMID: , PMID: 35757712 PMC9226890

[3] Lee HJ Kim M . Challenges and future trends in the treatment of psoriasis. Int J Mol Sci. (2023) 24. doi: 10.3390/ijms241713313, PMID: , PMID: 37686119 PMC10487560

[4] Schön MP Wilsmann-Theis D . Current developments and perspectives in psoriasis. J Dtsch Dermatol Ges. (2023) 21:363–72. doi: 10.1111/ddg.15033, PMID: , PMID: 37016915

[5] Kan J Chen Q Tao Q Wu L Wang D Jiang Z . Prospective evaluation of cardiovascular risk and mortality in patients with psoriasis: An American population-based study. Exp Dermatol. (2024) 33:e15010. doi: 10.1111/exd.15010, PMID: , PMID: 38284207

[6] Si Z Zhao H Ying J . Interaction effect of psoriasis and cancer on the risk of all-cause mortality: A prospective cohort study of NHANES data. Indian J Dermatol. (2024) 69:317–27. doi: 10.4103/ijd.ijd_1095_23, PMID: , PMID: 39296686 PMC11407579

[7] Mehta H Narang T Dogra S Handa S Hatwal J Batta A . Cardiovascular considerations and implications for treatment in psoriasis: an updated review. Vasc Health Risk Manage. (2024) 20:215–29. doi: 10.2147/vhrm.S464471, PMID: , PMID: 38745849 PMC11093123

[8] Koo J Marangell LB Nakamura M Armstrong A Jeon C Bhutani T . Depression and suicidality in psoriasis: review of the literature including the cytokine theory of depression. J Eur Acad Dermatol Venereol. (2017) 31:1999–2009. doi: 10.1111/jdv.14460, PMID: , PMID: 28681405

[9] Moher D Liberati A Tetzlaff J Altman DG . Preferred reporting items for systematic reviews and meta-analyses: the PRISMA statement. PloS Med. (2009) 6:e1000097. doi: 10.1371/journal.pmed.1000097, PMID: , PMID: 19621072 PMC2707599

[10] Stang A . Critical evaluation of the Newcastle-Ottawa scale for the assessment of the quality of nonrandomized studies in meta-analyses. Eur J Epidemiol. (2010) 25:603–5. doi: 10.1007/s10654-010-9491-z, PMID: , PMID: 20652370

[11] Wang M Pan H Zhai Y Li H Huang L Xie Z . Bidirectional association between rheumatoid arthritis and chronic obstructive pulmonary disease: a systematic review and meta-analysis. Front Immunol. (2024) 15:1494003. doi: 10.3389/fimmu.2024.1494003, PMID: , PMID: 39687614 PMC11647564

[12] Xie W Wang Y Xiao S Qiu L Yu Y Zhang Z . Association of gestational diabetes mellitus with overall and type specific cardiovascular and cerebrovascular diseases: systematic review and meta-analysis. Bmj. (2022) 378:e070244. doi: 10.1136/bmj-2022-070244, PMID: , PMID: 36130740 PMC9490552

[13] Zhao H Wu J Wu Q . Synergistic impact of psoriasis and hypertension on all-cause mortality risk: A prospective cohort study. PloS One. (2024) 19:e0306048. doi: 10.1371/journal.pone.0306048, PMID: , PMID: 38968326 PMC11226118

[14] Svedbom A Dalén J Mamolo C Cappelleri JC Mallbris L Petersson IF . Increased cause-specific mortality in patients with mild and severe psoriasis: a population-based Swedish register study. Acta Derm Venereol. (2015) 95:809–15. doi: 10.2340/00015555-2095, PMID: , PMID: 25766866

[15] Stern RS Huibregtse A . Very severe psoriasis is associated with increased noncardiovascular mortality but not with increased cardiovascular risk. J Invest Dermatol. (2011) 131:1159–66. doi: 10.1038/jid.2010.399, PMID: , PMID: 21248765

[16] Skov L Thomsen SF Kristensen LE Dodge R Hedegaard MS Kjellberg J . Cause-specific mortality in patients with psoriasis and psoriatic arthritis. Br J Dermatol. (2019) 180:100–7. doi: 10.1111/bjd.16919, PMID: , PMID: 29947129

[17] Semenov YR Herbosa CM Rogers AT Huang A Kwatra SG Cohen B . Psoriasis and mortality in the United States: data from the national health and nutrition examination survey. J Am Acad Dermatol. (2021) 85:396–403. doi: 10.1016/j.jaad.2019.08.011, PMID: , PMID: 31415837

[18] Prodanovich S Kirsner RS Kravetz JD Ma F Martinez L Federman DG . Association of psoriasis with coronary artery, cerebrovascular, and peripheral vascular diseases and mortality. Arch Dermatol. (2009) 145:700–3. doi: 10.1001/archdermatol.2009.94, PMID: , PMID: 19528427

[19] Pezzolo E Ciampichini R Cazzaniga S Sampietro G Zucchi A Naldi L . Psoriasis severity matters when dealing with all-cause mortality in psoriasis patients: a record linkage analysis in Northern Italy. Arch Dermatol Res. (2021) 313:255–61. doi: 10.1007/s00403-020-02101-1, PMID: , PMID: 32627048

[20] Mehta NN Azfar RS Shin DB Neimann AL Troxel AB Gelfand JM . Patients with severe psoriasis are at increased risk of cardiovascular mortality: cohort study using the General Practice Research Database. Eur Heart J. (2010) 31:1000–6. doi: 10.1093/eurheartj/ehp567, PMID: , PMID: 20037179 PMC2894736

[21] Lee MS Yeh YC Chang YT Lai MS . All-cause and cause-specific mortality in patients with psoriasis in Taiwan: A nationwide population-based study. J Invest Dermatol. (2017) 137:1468–73. doi: 10.1016/j.jid.2017.01.036, PMID: , PMID: 28257796

[22] Kong X Wang W . Synergistic effect of psoriasis and metabolic syndrome on risk of all-cause and cardiovascular mortality in US adults: a nationwide cohort study. Clin Exp Dermatol. (2024) 50:113–9. doi: 10.1093/ced/llae340, PMID: , PMID: 39152784

[23] Iskandar IYK Chen TC Chen LC Lee MS Yang YY Wang TC . Incidence, prevalence, and mortality of people with psoriasis and psoriatic arthritis in Taiwan: A nationwide cohort study. Acta Derm Venereol. (2022) 102:adv00807. doi: 10.2340/actadv.v102.1962, PMID: , PMID: 36065746 PMC9677272

[24] Gelfand JM Troxel AB Lewis JD Kurd SK Shin DB Wang X . The risk of mortality in patients with psoriasis: results from a population-based study. Arch Dermatol. (2007) 143:1493–9. doi: 10.1001/archderm.143.12.1493, PMID: , PMID: 18086997

[25] Dai YX Hsu MC Hu HY Chang YT Chen TJ Li CP . The risk of mortality among psoriatic patients with varying severity: A nationwide population-based cohort study in Taiwan. Int J Environ Res Public Health. (2018) 15. doi: 10.3390/ijerph15122622, PMID: , PMID: 30467301 PMC6313446

[26] Chen TC Wang TC Yiu ZZN Lee MS Chen LC Chan KA . Risk of serious infection and infection mortality in patients with psoriasis: A nationwide cohort study using the Taiwan National Health Insurance claims database. J Eur Acad Dermatol Venereol. (2024) 38:136–44. doi: 10.1111/jdv.19466, PMID: , PMID: 37611288

[27] Ahlehoff O Gislason GH Charlot M Jørgensen CH Lindhardsen J Olesen JB . Psoriasis is associated with clinically significant cardiovascular risk: a Danish nationwide cohort study. J Intern Med. (2011) 270:147–57. doi: 10.1111/j.1365-2796.2010.02310.x, PMID: , PMID: 21114692

[28] Abuabara K Azfar RS Shin DB Neimann AL Troxel AB Gelfand JM . Cause-specific mortality in patients with severe psoriasis: a population-based cohort study in the U. K. Br J Dermatol. (2010) 163:586–92. doi: 10.1111/j.1365-2133.2010.09941.x, PMID: , PMID: 20633008 PMC2966545

[29] Zhou T Wu J Wang Y Gao Y Cheng K . Weight-adjusted waist index, psoriasis, and all-cause mortality: findings from the NHANES 2003-2006 and 2009-2014. Clin Cosmet Investig Dermatol. (2025) 18:7–18. doi: 10.2147/CCID.S497128, PMID: , PMID: 39781099 PMC11708201

[30] Springate DA Parisi R Kontopantelis E Reeves D Griffiths CE Ashcroft DM . Incidence, prevalence and mortality of patients with psoriasis: a U.K. population-based cohort study. Br J Dermatol. (2017) 176:650–8. doi: 10.1111/bjd.15021, PMID: , PMID: 27579733 PMC5363241

[31] Dhana A Yen H Yen H Cho E . All-cause and cause-specific mortality in psoriasis: A systematic review and meta-analysis. J Am Acad Dermatol. (2019) 80:1332–43. doi: 10.1016/j.jaad.2018.12.037, PMID: , PMID: 30590074

[32] Piaserico S Orlando G Messina F . Psoriasis and cardiometabolic diseases: shared genetic and molecular pathways. Int J Mol Sci. (2022) 23. doi: 10.3390/ijms23169063, PMID: , PMID: 36012327 PMC9409274

[33] Kalb RE Fiorentino DF Lebwohl MG Toole J Poulin Y Cohen AD . Risk of serious infection with biologic and systemic treatment of psoriasis: results from the psoriasis longitudinal assessment and registry (PSOLAR). JAMA Dermatol. (2015) 151:961–9. doi: 10.1001/jamadermatol.2015.0718, PMID: , PMID: 25970800

[34] Syed MN Shin DB Wan MT Winthrop KL Gelfand JM . The risk of respiratory tract infections in patients with psoriasis treated with interleukin 23 pathway-inhibiting biologics: A meta-estimate of pivotal trials relevant to decision making during the COVID-19 pandemic. J Am Acad Dermatol. (2020) 83:1523–6. doi: 10.1016/j.jaad.2020.06.1014, PMID: , PMID: 32622891 PMC7331500

[35] Biazus Soares G Mahmoud O Yosipovitch G Mochizuki H . The mind-skin connection: A narrative review exploring the link between inflammatory skin diseases and psychological stress. J Eur Acad Dermatol Venereol. (2024) 38:821–34. doi: 10.1111/jdv.19813, PMID: , PMID: 38311707

[36] Mateu-Arrom L Puig L . Genetic and epigenetic mechanisms of psoriasis. Genes (Basel). (2023) 14. doi: 10.3390/genes14081619, PMID: , PMID: 37628670 PMC10454222

[37] Blackstone B Patel R Bewley A . Assessing and improving psychological well-being in psoriasis: considerations for the clinician. Psoriasis (Auckl). (2022) 12:25–33. doi: 10.2147/PTT.S328447, PMID: , PMID: 35371967 PMC8965012

[38] Kaushik SB Lebwohl MG . Psoriasis: Which therapy for which patient: Psoriasis comorbidities and preferred systemic agents. J Am Acad Dermatol. (2019) 80:27–40. doi: 10.1016/j.jaad.2018.06.057, PMID: , PMID: 30017705

