# Supplementary material for: All-cause and cause-specific mortality in psoriasis patients: a systematic review and meta-analysis
Source: Front Immunol. 2025 Jul 24;16:1610499. doi: 10.3389/fimmu.2025.1610499 (PMC12328333; doi:10.3389/fimmu.2025.1610499)
Supplement: Supplementary file 1 [file DataSheet1.docx]

**Supplementary Material Figures**

Supplementary Material Figure 1. Sensitivity analysis for the risk of all-cause mortality in psoriasis

Supplementary Material Figure 2. Sensitivity analysis for the risk of cardiovascular mortality in psoriasis

Supplementary Material Figure. 3 Sensitivity analysis for the risk of infection mortality in psoriasis

Supplementary Material Figure 4. Sensitivity analysis for the risk of neoplasm mortality in psoriasis

Supplementary Material Figure 5. Sensitivity analysis for the risk of suicide mortality in psoriasis
