# Supplementary material for: All-cause and cause-specific mortality in psoriasis patients: a systematic review and meta-analysis
Source: Front Immunol. 2025 Jul 24;16:1610499. doi: 10.3389/fimmu.2025.1610499 (PMC12328333; doi:10.3389/fimmu.2025.1610499)
Supplement: Supplementary file 2 [file Table1.docx]

Supplementary Material Table 1. Search strategy

| Databased | No. | Content | Result |
| --- | --- | --- | --- |
| PubMed | #1 | "Psoriasis"[MeSH Terms] Sort by: Most Recent | 51,655 |
|  | #2 | "Psoriasis"[Title/Abstract] Sort by: Most Recent | 56,016 |
|  | #3 | "Psoriasis"[MeSH Terms] OR "Psoriasis"[Title/Abstract] Sort by: Most Recent | 68,720 |
|  | #4 | "Mortality"[MeSH Terms] Sort by: Most Recent | 438,779 |
|  | #5 | "mortalit*"[Title/Abstract] OR "death*"[Title/Abstract] OR "fatality rate*"[Title/Abstract] Sort by: Most Recent | 2,017,762 |
|  | #6 | "Mortality"[MeSH Terms] OR "mortalit*"[Title/Abstract] OR "death*"[Title/Abstract] OR "fatality rate*"[Title/Abstract] Sort by: Most Recent | 2,231,011 |
|  | #7 | "Risk"[MeSH Terms] Sort by: Most Recent | 1,464,554 |
|  | #8 | "Risk"[Title/Abstract] Sort by: Most Recent | 3,114,331 |
|  | #9 | "Risk"[MeSH Terms] OR "Risk"[Title/Abstract] Sort by: Most Recent | 3,643,376 |
|  | #10 | ("Psoriasis"[MeSH Terms] OR "Psoriasis"[Title/Abstract]) AND ("Mortality"[MeSH Terms] OR ("mortalit*"[Title/Abstract] OR "death*"[Title/Abstract] OR "fatality rate*"[Title/Abstract])) AND ("Risk"[MeSH Terms] OR "Risk"[Title/Abstract]) Sort by: Most Recent | 701 |
| Embase | #1 | 'psoriasis'/exp | 128963 |
|  | #2 | 'psoriasis':ab,ti | 85457 |
|  | #3 | #1 OR #2 | 135973 |
|  | #4 | 'mortality'/exp | 1551212 |
|  | #5 | 'mortalit*':ab,ti OR 'death*':ab,ti OR 'fatality rate*':ab,ti | 2911723 |
|  | #6 | #4 OR #5 | 3305612 |
|  | #7 | 'risk'/exp | 3397579 |
|  | #8 | 'risk':ab,ti | 4460118 |
|  | #9 | #7 OR #8 | 5451672 |
|  | #10 | #3 AND #6 AND #9 | 2460 |
| Cochran Library | #1 | Psoriasis | 10392 |
|  | #2 | (Psoriasis):ti,ab,kw | 10247 |
|  | #3 | #1 OR #2 | 10392 |
|  | #4 | Mortality | 128934 |
|  | #5 | (Mortalit*):ti,ab,kw OR (Death*):ti,ab,kw OR (Fatality Rate*):ti,ab,kw | 187670 |
|  | #6 | #4 OR #5 | 191384 |
|  | #7 | Risk | 320802 |
|  | #8 | (Risk):ti,ab,kw | 312959 |
|  | #9 | #7 OR #8 | 320802 |
|  | #10 | #3 AND #6 AND #9 | 154 |
